# Ameliorative Effect of Fisetin on Cisplatin-Induced Nephrotoxicity in Rats via Modulation of NF-κB Activation and Antioxidant Defence

**DOI:** 10.1371/journal.pone.0105070

**Published:** 2014-09-03

**Authors:** Bidya Dhar Sahu, Anil Kumar Kalvala, Meghana Koneru, Jerald Mahesh Kumar, Madhusudana Kuncha, Shyam Sunder Rachamalla, Ramakrishna Sistla

**Affiliations:** 1 Medicinal Chemistry and Pharmacology Division, CSIR-Indian Institute of Chemical Technology (IICT), Hyderabad, Andhra Pradesh, India; 2 Department of Pharmacology and Toxicology, National Institute of Pharmaceutical Education and Research (NIPER), Hyderabad, Andhra Pradesh, India; 3 Animal House, CSIR-Centre for Cellular and Molecular Biology (CCMB), Hyderabad, Andhra Pradesh, India; 4 Faculty of Pharmacy, Osmania University, Hyderabad, Andhra Pradesh, India; National Institutes of Health, United States of America

## Abstract

Nephrotoxicity is a dose-dependent side effect of cisplatin limiting its clinical usage in the field of cancer chemotherapy. Fisetin is a bioactive flavonoid with recognized antioxidant and anti-inflammatory properties. In the present study, we investigated the potential renoprotective effect and underlying mechanism of fisetin using rat model of cisplatin-induced nephrotoxicity. The elevation in serum biomarkers of renal damage (blood urea nitrogen and creatinine); degree of histopathological alterations and oxidative stress were significantly restored towards normal in fisetin treated, cisplatin challenged animals. Fisetin treatment also significantly attenuated the cisplatin-induced IκBα degradation and phosphorylation and blocked the NF-κB (p65) nuclear translocation, with subsequent elevation of pro-inflammatory cytokine, TNF-α, protein expression of iNOS and myeloperoxidase activities. Furthermore, fisetin markedly attenuated the translocation of cytochrome c protein from the mitochondria to the cytosol; decreased the expression of pro-apoptotic proteins including Bax, cleaved caspase-3, cleaved caspase-9 and p53; and prevented the decline of anti-apoptotic protein, Bcl-2. The cisplatin-induced mRNA expression of NOX2/gp91phox and NOX4/RENOX and the NADPH oxidase enzyme activity were also significantly lowered by fisetin treatment. Moreover, the evaluated mitochondrial respiratory enzyme activities and mitochondrial antioxidants were restored by fisetin treatment. Estimation of platinum concentration in kidney tissues revealed that fisetin treatment along with cisplatin did not alter the cisplatin uptake in kidney tissues. In conclusion, these findings suggest that fisetin may be used as a promising adjunct candidate for cisplatin use.

## Introduction

Though recent investigations find the new generation of platinum-based cytotoxic agents, cisplatin (cis-Diamminedichloroplatinum II, CDDP) remains a highly effective and widely used anti-neoplastic drug against various solid tumors, including endometrial, testicular, ovarian, breast, bladder, head, neck and lung cancer [1]. Despite being a potent anticancer drug, cisplatin elicits dose and duration dependent nephrotoxicity limiting its clinical utility in 25–35% of hospitalized patients undergoing chemotherapy [2]. Owing to its potent and wide range of therapeutic benefit against various malignancies, establishment of a new adjunct therapeutic strategy which ameliorates the severity of cisplatin elicited toxicity in the field of cancer research is therefore warranted.

Recent studies in molecular mechanism of chemotherapy induced toxicity have revealed that cisplatin-induced nephrotoxicity is multifactorial and numerous signalling pathways are involved in. Studies demonstrated that accumulation of cisplatin in renal tubular cells is five times more in comparison with other tissues and is considered as one of the prime reason for cisplatin-induced nephrotoxicity [1]. Many studies, including ours, have demonstrated that oxidative stress due to impaired antioxidant status and/or excess generation of free radicals, in particular superoxide, due to cisplatin-induced renal NADPH oxidase NOX4 (RENOX) and phagocyte NADPH oxidase (NOX2/gp91phox) over expression is involved in such deleterious effects [2], [3]. Evidences from *in vitro* and *in vivo* studies also have demonstrated that cisplatin induces apoptosis and necrosis of renal tubular cells through activation of both intrinsic and extrinsic mitochondrial pathways [4], [5]. In addition, studies also document involvement and activation of p53 mediated pro-apoptotic molecules in cisplatin-induced nephrotoxicity [6], [7]. Moreover, activation of pro-inflammatory pathways (TNF-α, NF-κB) and infiltration of inflammatory cells are the other crucial mechanisms involved in cisplatin-induced nephrotoxicity [8]. A growing body of evidence also suggests the mitochondrial dysfunction, generation of mitochondrial reactive oxygen species (ROS) and impairment of mitochondrial antioxidant activities trigger the deleterious cascade of renal tissue injury in cisplatin administered rats [9], [10].

Fisetin (3, 7, 3′, 4′-tetrahydroxyflavone) is a bioactive polyphenolic flavonoid, commonly found in many fruits and vegetables such as strawberries, apples, persimmons, onions and cucumbers [11]. In a recent review, fisetin was found to be one of the potent antioxidant flavonoid among the evaluated flavonoids [12]. Fisetin has been shown to posses both direct intrinsic antioxidant as well as indirect antioxidant effects by increasing levels of reduced glutathione in *in vitro* neuronal cells [13]. It exerts multiple beneficial pharmacological activities such as anti-inflammatory, anticancer, hypolipidemic and in rheumatoid arthritis [14]. Recent investigations suggests that fisetin attenuates migration and invasion of cervical cancer cells [15]; inhibits allergic airway inflammation [16], prevents adipocyte differentiation [17], prevents hepatosteatosis [18], attenuates complications of diabetes [19], counters osteoporosis [14] and exerts neuroprotection in cerebral ischemic condition [13]. In addition, fisetin is a potent natural anticancer agent. A study conducted by Tripathi et al. [20] demonstrated that the fisetin treatment along with cisplatin increased the cytotoxic effect of cisplatin by four-fold. However, the effect of fisetin on cisplatin-induced nephrotoxicity has not been evaluated. Based on the aforementioned facts and in continuation with the study conducted by Tripathi et al. [20], the present investigation was designed to evaluate the effect of fisetin on kidney tissues of cisplatin treated rats. We report that fisetin pre-treatment significantly ameliorates cisplatin-induced renal impairments, histopathological alterations and restores antioxidant and mitochondrial respiratory enzyme activities in kidney tissues. Furthermore, the results of the present study at molecular and cellular level revealed that fisetin significantly attenuates cisplatin-induced renal NOX4/RENOX and NOX2/gp91phox expression, apoptosis related protein expressions, modulates NF-κB activation and subsequent inflammation in kidney tissues.

## Materials and Methods

### Ethics statement and experimental animals

This study was carried out in strict accordance with recommendations on use of experiment animals according to Committee for the Purpose of Control and Supervision of Experiments on Animals (CPCSEA), Govt. of India for safe use and care of experimental animals. The protocol was approved by the committee called “Institutional Animal Ethics Committee (IAEC)” of Indian Institute of Chemical technology (IICT), Hyderabad, India (Approval No: IICT/PHARM/SRK/26/08/2013/09). All efforts were made to minimize sufferings. Male Sprague-Dawley rats weighing between 180 and 200 g were procured from National Institute Nutrition (NIN), Hyderabad, India and were housed under controlled environmental conditions (12 h light: 12 h dark cycle, 22±2°C temperature and 55±15% relative humidity). Animals were acclimatized for 7 days and allowed free access of food and water at all times.

### Drugs and chemicals

Cisplatin, fisetin (purity: ≥98%), superoxide dismutase (SOD) assay kit, cytochrome c oxidase assay kit, o-dianisidine, MTT [3-(4, 5-dimethylthiazol-2-yl)-2, 5-diphenyltetrazolium bromide, β-nicotinamide adenine dinucleotide 3-phosphate reduced form (NADPH), lucigenin, β-nicotinamide adenine dinucleotide hydrate (NADH), succinic acid, 2, 6-dichlorophenolindophenol (DCIP), reduced glutathione (GSH), catalase (CAT), oxidized glutathione (GSSG), 5, 5-dithio-bis (2-nitrobenzoic acid) (DTNB), Bradford reagent, 2-thiobarbituric acid (TBA) and cytochrome c were purchased from Sigma-Aldrich Co, St Louis, MO, USA. Bicinchoninic acid (BCA) protein assay kit was purchased from Pierce Biotechnology, Rockford, IL, USA. NF-κB (p^65^) transcription factor assay kit was obtained from Cayman Chemical Company, Ann Arbor, MI. Rat TNF-α (BD OptEIA) and IL-6 (BD OptEIA) ELISA kits were obtained from BD Bioscience, San Diego, CA, USA. All other chemicals were of analytical grade.

### Experimental design

**Figure 1:**
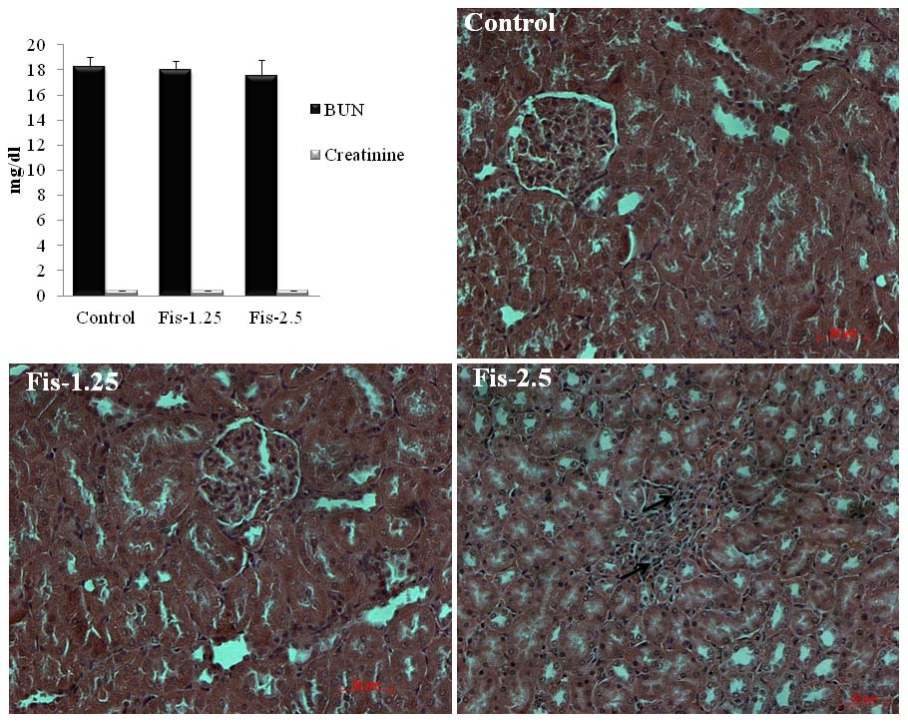
Effect of fisetin itself on serum renal function parameters and kidney histology. Intraperitoneal administration of fisetin at two different doses i.e. 1.25 mg/kg and 2.5 mg/kg body weight for 7 consecutive days showing normal BUN (blood urea nitrogen) and serum creatinine levels compared to vehicle control rats. Histopathological examination of kidney tissues (X200 magnification, scale bar: 50 µm) from vehicle control (Control) and fisetin at 1.25 mg/kg (Fis-1.25) treated rats showing apparently normal histo-morphology. Histopathological findings from fisetin at 2.5 mg/kg treated rats (Fis-2.5) showing moderate infiltration of inflammatory cells (black arrow) in kidney tissues.

Prior to initiation of main experiment, a pilot study was conducted to confirm the safety and effect of fisetin alone on renal tissues. Based on the previous literature [20], fisetin at two different doses i.e. 1.25 and 2.5 mg/kg was selected and administered intraperitoneally for 7 consecutive days. On 8^th^ day, blood samples were collected through retro-orbital plexus and serum was separated for the estimation of serum specific renal injury (BUN and creatinine) biomarkers. Kidneys were dissected and processed for histopathological evaluation. From this pilot study, it was observed that serum levels of BUN and creatinine were not showing statistically significant (p>0.05) differences between vehicle control, fisetin at 1.25 mg/kg and fisetin at 2.5 mg/kg administered group of rats (Figure 1). Histopathological findings of kidneys from the rats treated with vehicle (Control) and fisetin at a dose of 1.25 mg/kg (Fis-1.25) revealed normal kidney histo-morphology. To our surprise, a moderate infiltration of inflammatory cells was observed in kidney tissues of rats administered with 2.5 mg/kg fisetin (Fis-2.5) (Figure 1). Based on this finding, we further evaluated the effect of fisetin alone on heart and liver tissues. Cardiac injury (serum levels of CK-MB and LDH, and histopathology of heart, Figure S1) and hepatic injury (serum levels of SGOT and SGPT, and histopathology of liver, Figure S2) markers revealed that fisetin administration did not produce any significant alterations in the heart and liver tissue compared to vehicle control rats. Based on the above findings, fisetin at two different doses i.e. 0.625 and 1.25 mg/kg was selected for the main study. Fisetin, being poorly soluble in aqueous medium, was dissolved in PEG200/DMSO (7∶3; v∶v) and was administered intraperitoneally to the rats once in a day for 7 consecutive days [21]. Same vehicle system was administered to control rats.

In the main study, forty animals were randomly divided into 5 groups containing 8 rats in each. Group I (Vehicle control, Control), animals were administered with 40 µl of PEG200/DMSO (7∶3) intraperitoneally once daily for 7 consecutive days and a single intraperitoneal injection of normal saline on 3^rd^ day. In group II (Fisetin control, Fis), animals were intraperitoneally administered with fisetin [1.25 mg/kg dissolved in PEG200/DMSO (7∶3)] once daily for 7 consecutive days and a single intraperitoneal injection of normal saline on 3^rd^ day. In group III (Cisplatin control, Cis), animals were intraperitoneally administered with 40 µl of PEG200/DMSO (7∶3) once daily for 7 consecutive days and a single intraperitoneal injection of cisplatin (5 mg/kg dissolved in normal saline) on 3^rd^ day. In group IV (Cis+Fis-0.625 mg), animals were intraperitoneally administered with 40 µl of 0.625 mg/kg of fisetin [dissolved in PEG200/DMSO (7∶3)] once daily for 7 consecutive days and a single intraperitoneal injection of cisplatin (5 mg/kg dissolved in normal saline) on 3^rd^ day. In group V (Cis+Fis-1.25 mg), animals were intraperitoneally administered with 40 µl of 1.25 mg/kg of fisetin [dissolved in PEG200/DMSO (7∶3)] once daily for 7 consecutive days and a single intraperitoneal injection of cisplatin (5 mg/kg dissolved in normal saline) on 3^rd^ day. At the end of the experiment (i.e. on 8^th^ day), body weight of all animals was recorded. Blood samples were collected from all the experimental animals and serum was separated. The animals were euthanized with CO_2_ asphyxiation; kidney tissues were isolated, relative weights of kidneys (i.e kidney to body weight ratio normalized to 100 g body weight of animals) were determined and then stored at −80°C for further study.

### Estimation of serum blood urea nitrogen (BUN) and creatinine

To assess the renal failure, serum levels of BUN and creatinine were estimated using auto blood analyzer (Siemens, Dimension Xpand^plus^, USA) by employing BUN and creatinine assay kits (Siemens, India).

### Histopathology of kidney tissues

For histopathological evaluation of kidney tissues, tissue samples were fixed in 10% neutral buffered formalin and were processed for embedding in paraffin wax. Thin sections (5 µm) of the kidney tissues were cut using a microtome (Leica, Bensheim, Germany) and were stained with hematoxylin and eosin (H & E). The sections were examined for histopathological changes under light microscopy using Zeiss microscope (Axioplan 2 Imaging, Axiovision software).

### Tissue preparation and biochemical estimations

A 10% kidney tissues homogenate was prepared with phosphate buffer saline (50 mM, pH 7.4) containing 1% protease inhibitor cocktail (Sigma-Aldrich Co, St Louis, MO, USA) using Teflon homogenizer. Homogenates were centrifuged at 14000 g for 45 min and supernatant obtained was used for estimation of various biochemical parameters. The kidney tissues content of reduced glutathione (GSH) [22], glutathione reductase (GR) [23], glutathione S-transferase (GST) [24], catalase (CAT) [25], superoxide dismutase (SOD) (SOD assay kit, Sigma–Aldrich Co., St. Louis, MO, USA), NAD (P) H: quinine oxidoreductase 1 (NQO1) [26], vitamin C [27] and thiobarbituric acid reactive substances (TBARS) as an index of lipid peroxidation [28] were estimated as described in earlier literatures. Total protein content in kidney tissues homogenate was estimated using Bradford reagent (Sigma-Aldrich Co, St Louis, MO, USA) and bovine serum albumin (BSA) as standard.

### Isolation of mitochondrial and cytosolic fraction from kidney tissues

The mitochondrial and cytosolic fractions of kidney tissues were isolated as described by Wei et al. [29]. Briefly, a portion of the fresh kidney tissue was homogenized in ice-cold isolation buffer [270 mM Sucrose, 1 mM EGTA and 5 mM Tris (pH 7.4)]. The homogenates were centrifuged at 600 g for 10 min at 4°C to remove cell debris and nuclei. The obtained supernatant was once again centrifuged at 10000 g for 10 min at 4°C to collect the mitochondrial fraction in the pellets. The supernatant fractions were centrifuged again at 100000 g for 1 h (Thermo Scientific Sorvall Discovery M150 SE Ultra centrifuge) to collect the cytosolic fraction. Protein concentration in mitochondrial and cytosolic fraction was determined using Bradford reagent and bovine serum albumin (BSA) as standard.

### Estimation of mitochondrial respiratory enzyme activities

The mitochondrial respiratory enzyme activities such as NADH dehydrogenase [30], succinate dehydrogenase [31] and cytochrome c oxidase (cytochrome c oxidase assay kit, Sigma-Aldrich Co, St Louis, MO, USA) were estimated as reported in earlier standard procedure. In addition, mitochondrial redox activity was assessed by incubating the isolated mitochondria with MTT solution (5 mg MTT/ml of 50 mM phosphate buffer saline, pH 7.4) for 3 h at 37°C [32]. Depending upon the mitochondrial intactness, formazan crystals thus formed were solubilised in dimethyl sulfoxide and optical density was recorded at 580 nm.

### Estimation of mitochondrial antioxidant and lipid peroxidation parameters

The mitochondrial non-enzymatic antioxidant, GSH [22] and enzymatic antioxidants i.e. GR [23], GST [24], CAT [25] and SOD (SOD assay kit, Sigma-Aldrich Co, St Louis, MO, USA) activities were analysed as described in earlier methods. Kidney tissue mitochondrial TBARS content was determined, as an index of lipid peroxidation, as described in earlier literature [28].

### Estimation of TNF-α and IL-6 in kidney tissues

For estimation of pro-inflammatory cytokines in kidney tissues, a 10% tissue homogenate was prepared with phosphate buffer saline (50 mM, pH 7.4) containing 1% protease inhibitor cocktail (Sigma-Aldrich Co, St Louis, MO, USA). Then, the homogenates were centrifuged at 4000 g for 20 min and the supernatant obtained were used for the estimation of TNF-α and IL-6 using rat TNF-α (BD OptEIA) and IL-6 (BD OptEIA) ELISA kits (BD Bioscience, San Diego, CA, USA) respectively. The concentrations of TNF-α and IL-6 in kidney tissues were expressed as pg/mg protein.

### Estimation of myeloperoxidase (MPO) activity in kidney tissues

Myeloperoxidase activity was estimated as described in previously published procedure [33]. Briefly, a 10% kidney tissues homogenate was prepared using ice-cold 50 mM potassium phosphate buffer (pH 6) containing 0.5% hexadecyltrimethylammonium bromide (HTAB) and 10 mM EDTA. Homogenates were subjected to one cycle of freeze and thaw, followed by brief sonication. Then the homogenates were centrifuged at 13100 g for 20 min. The supernatant obtained was used for estimation of MPO activity using 0.167 mg/ml of o-dianisidine hydrochloride and 0.0005% hydrogen peroxide at 460 nm. The MPO activity was expressed as U/g of tissue.

### Estimation of NADPH oxidase activity in kidney tissues

The activity of NADPH oxidase was estimated according to Oh et al [34]. Briefly, a 10% kidney tissues homogenate were prepared using ice-cold phosphate buffer saline (50 mM, pH 7.4). The homogenates were centrifuged at 4000 g for 15 min at 4°C to remove any cellular debris. The protein concentration in homogenates was estimated using Bradford reagent and BSA as standard. Around 50 µg protein of kidney homogenates was incubated with a reaction mixture contained 1 mM ethylene glycol tetra acetic acid and 5 µM lucigenin in 50 mM phosphate buffer, pH 7.0. The reaction was started by the addition of 50 µM NADPH to the reaction mixture and the activity was estimated by recording the luminescence for 5 min at every 31 s interval. No activity could be measured in the absence of NADPH. Relative luminescence unit (RLU) was calculated and was expressed as fold increase over control.

### RNA isolation and quantitative Real-time PCR

Total RNA was isolated from frozen kidney tissues using TRI reagent (Sigma-Aldrich Co, St Louis, MO, USA) according to manufacturer's protocol. RNA concentration was quantified using NanoDrop 2000/2000c (Thermo Fisher Scientific, Wilmington, DE 19810 U.S.A). cDNA were synthesized using Thermo Scientific Verso cDNA kit according to manufacturers' instructions. NADPH oxidase subunits of mRNA (NOX2/gp91phox and NOX4/RENOX) expression were measured by quantitative real-time polymerase chain reaction (PCR) analysis using the StepOnePLUS (Applied Biosystems, USA) real-time PCR detection system. The reaction mixture of real-time PCR contained 100 ng 5′ and 3′ primer and 2 µl of cDNA product in Thermo Scientific DyNAmo ColorFlash SYBR Green qPCR Kit. All primers were synthesized by Geno Bioscience Pvt Ltd (India). The primers used for rat NOX2/gp91phox were (forward) 5′-CCCTTTGGTACAGCCAGTGAAGAT-3′ and (reverse) 5′-CAATCCCAGCTCCCACTAACATCA-3′, rat NOX4/RENOX (forward) 5′-GGATCACAGAAGGTCCCTAGCAG-3′ and (reverse) 5′-GCAGCTACATGCACACCTGAGAA-3′, and those used for rat GAPDH were (forward) 5′-TCAAGAAGGTGGTGAAGCAG-3′ and (reverse) 5′-AGGTGGAAGAATGGGAGTTG-3′. The real-time PCR results for the mRNA levels of each gene were normalized to GAPDH levels. Relative mRNA expression was quantified using the ΔΔCt method. Results were expressed as fold change.

### Nuclear, cytoplasmic and total protein extraction

Nuclear, cytoplasmic and total protein extracts from kidney tissues of different experimental groups were prepared as described in our previously published literature [35]. Protein concentrations in all extracts were determined using Bicinchoninic acid (BCA) assay kit (Pierce Biotechnology, Rockford, IL, USA) against bovine serum albumin (BSA) as standard and stored them at −80°C till further analysis.

### Antibodies and immunoblot analysis

Antibodies against cleaved caspase 3, cleaved caspase 9, Bax, Bcl-2, cytochrome c, p53, NF-κB (p65), IκBα, phospho-IκBα, β-actin, lamin B and HRP-conjugated secondary anti-rabbit and anti-mouse antibodies were purchased from Cell Signaling Technology, Boston, MA. Antibody against iNOS was obtained from Sigma-Aldrich Co, St Louis, MO, USA. For detection of protein expression in renal cortex, an equal amount of protein (40 µg/lane) samples were resolved in 10% SDS-PAGE and electrophoretically transferred onto polyvinylidine difluoride membranes (PVDF, Pierce Biotechnology, Rockford, IL, USA) for immunoblot analysis. The following dilutions were made for primary antibody preparation for detection of total protein; monoclonal rabbit cleaved caspase 3 (1∶1000), monoclonal mouse cleaved caspase 9 (1∶1000), monoclonal rabbit Bax (1∶1000), monoclonal rabbit Bcl-2 (1∶1000), monoclonal mouse p53 (1∶1000), monoclonal rabbit iNOS (1∶500); for detection of nuclear protein, monoclonal rabbit NF-κB (p65) (1∶500); for detection of cytoplasmic protein, monoclonal mouse IκBα (1∶500) and monoclonal rabbit phospho-IκBα (1∶500) antibodies; HRP-conjugated anti-rabbit and anti-mouse secondary antibodies at a dilution of 1∶8000 were used. Similarly, mitochondrial and cytosolic fractions were used for detection of cytosolic translocation of cytochrome c (rabbit monoclonal, 1∶1000). β-actin (1∶1000) was used for equal loading of total, cytoplasmic and cytosolic proteins and Lamin B (1∶1000) was used for equal loading of nuclear proteins. Coomassie blue stain was used for equal loading of mitochondrial protein. The membranes were visualized with SuperSignal West Pico Chemiluminescent Substrate (Pierce Biotechnology, Rockford, IL, USA) and developed to X-ray film (Pierce Biotechnology, Rockford, IL, USA). Band intensities were quantified by using Image J software (NIH).

### NF-κB (p65) transcription assay

As per the manufacturer instructions, NF-κB (p^65^) transcription factor ELISA assay kit (Cayman Chemical Company, Ann Arbor, MI) was used to evaluate the NF-κB DNA-binding activity of nuclear samples extracted from kidney tissues of different experimental groups.

### Estimation of platinum in kidney tissues

To analyze the platinum content in kidney tissues, as described in earlier method, known quantity of kidney tissues were digested in concentrated nitric acid at a temperature of 40°C for 3 h under constant agitation [36]. The platinum concentration of digested samples were assessed by Inductively Coupled Plasma Mass Spectrometry (ICP-MS) method and expressed as µg/g of tissue.

### Statistical analysis

Experimental data was represented as Mean ± S.E.M. Graph Pad Prism software (version 5.0) was used for statistical analysis of the study. Significance difference was evaluated by performing one-way analysis of variance (ANOVA) with Dunnett's multiple comparison procedure; *p* values of less than 0.05 were regarded as significant.

## Results

### Effect of fisetin on cisplatin-induced renal injury parameters

**Figure 2:**
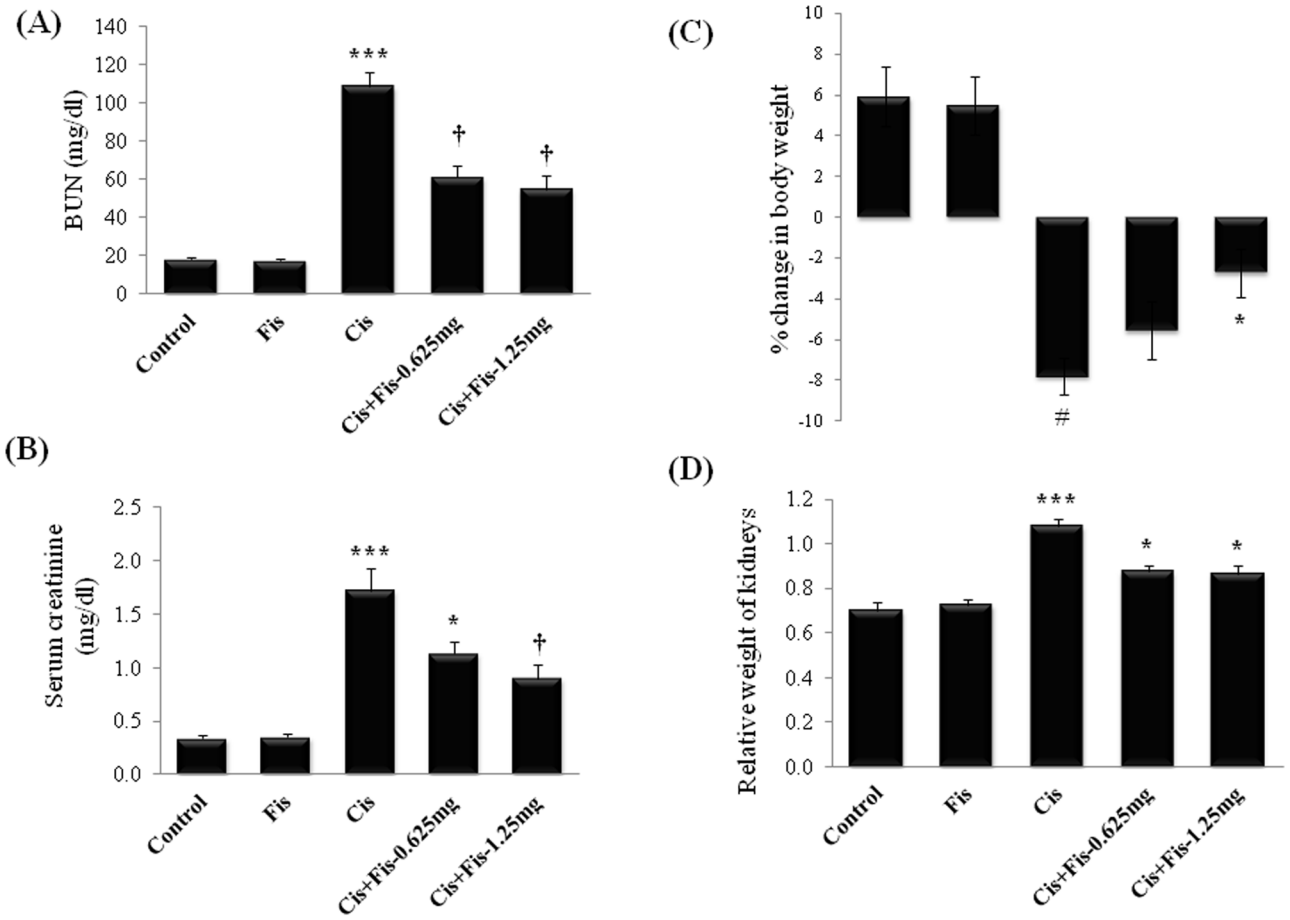
Effect of fisetin on cisplatin-induced changes in serum renal function parameters. (**A**) Blood urea nitrogen (BUN), (**B**) Creatinine, (**C**) Percentage change in body weight and (**D**) Relative weight of kidneys in different experimental groups. Fisetin was administered intraperitoneally at two different doses i.e. 0.625 mg/kg and 1.25 mg/kg for 7 consecutive days and a single dose of cisplatin (5 mg/kg, i.p) on 3^rd^ day. On 8^th^ day, serum levels of BUN and creatinine, percentage change in body weight and relative weight of kidneys were recorded. The results were expressed as mean ± S.E.M of 8 animals in each group. Where, Control, vehicle control; Fis, fisetin control; Cis, cisplatin control; Cis+Fis-0.625 mg, cisplatin plus fisetin (0.625 mg/kg) treated groups; Cis+Fis-1.25 mg, cisplatin plus fisetin (1.25 mg/kg) treated groups. ^*^p<0.05 and ^†^p<0.001vs cisplatin control group; ^#^p<0.05 and ^***^p<0.001 vs vehicle control group.

**Figure 3:**
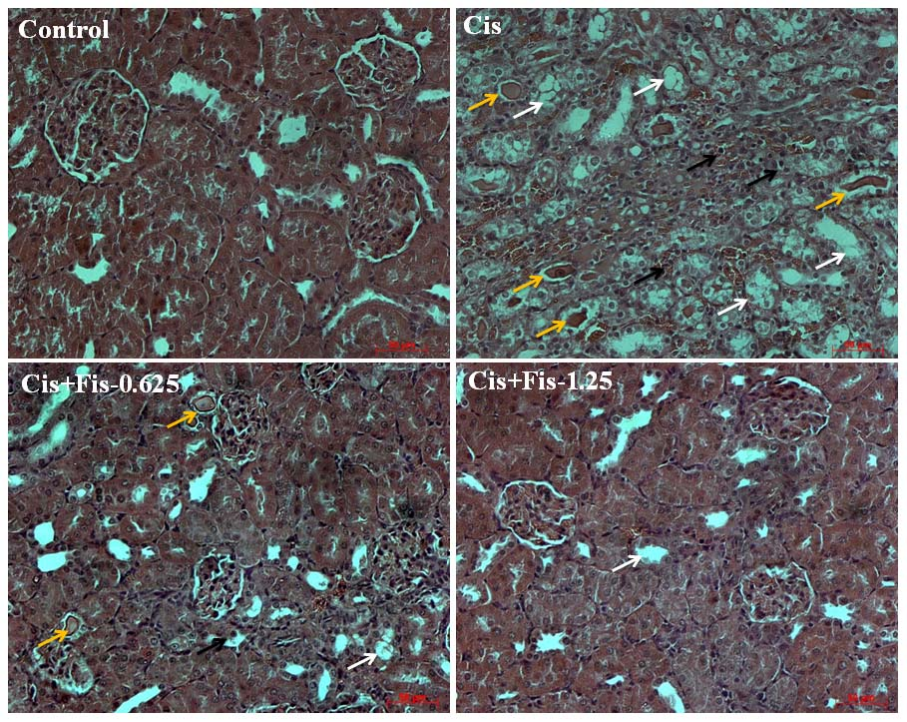
Histopathological observations of kidney tissues from rats treated with fisetin and cisplatin. Representative photomicrographs of kidney sections stained with Hematoxylin-eosin (H and E). Light microscopic examination of kidney sections from vehicle control rats (Control) showing normal glomeruli and tubules (X200 magnification, scale bar: 50 µm). Kidney tissue sections from cisplatin alone treated rats (Cis) showing severe tubular degeneration and necrosis (white arrow), infiltration of inflammatory cells (black arrow) and accumulation of homogenous eosinophilic casts (yellow arrow) in the lumen of the tubules (X200 magnification, scale bar: 50 µm). Kidney tissue sections from low dose of fisetin (0.625 mg/kg) plus cisplatin treated rats (Cis+Fis-0.625) showing recovery of tubular degeneration (white arrow), mild infiltration of inflammatory cells (black arrow) and decreased accumulation of eosinophilic casts in tubular lumen (yellow arrow) when compared to cisplatin alone treated rats (X200 magnification, scale bar: 50 µm). Kidney tissue sections from high dose of fisetin (1.25 mg/kg) plus cisplatin treated rats (Cis+Fis-1.25) showing predominantly normal renal histology with occasional degenerative changes (white arrow) when compared to cisplatin alone treated rats (X200 magnification, scale bar: 50 µm).

Cisplatin administration significantly (p<0.001) increased the BUN (Figure 2A) (from 18.25±0.53 mg/dl to 108.33±7.64 mg/dl) and creatinine (Figure 2B) (from 0.34±0.03 mg/dl to 1.72±0.21 mg/dl) levels when compared to vehicle control groups. Fisetin treatment at both the doses (0.625 and 1.25 mg/kg) along with cisplatin significantly attenuated the increase in BUN and creatinine levels when compared to cisplatin alone treated group. No mortality was observed in animals treated with cisplatin and/or fisetin during the study period. For calculation of percentage of body weight gain/loss, the day of cisplatin administration is considered as day 0 and average body weight of all animals was taken as 0%. After 5 days of cisplatin administration, body weight of all animals from each group was recorded and percentage of weight gain or loss was calculated. In vehicle control and fisetin (1.25 mg/kg) alone treated group, we observed a 5.9% and 5.5% body weight gain respectively, when compared to day 0 body weight of respective group animals. In cisplatin alone administered group of rats, we observed a 7.8% body weight loss when compared to day 0 body weight of same animals. Fisetin (1.25 mg/kg) treatment along with cisplatin significantly (p<0.05) attenuated the body weight loss when compared to cisplatin alone treated rats (Figure 2C). Similarly, relative weight of kidneys in cisplatin alone treated rats were significantly (p<0.001) increased compared to vehicle control group. Fisetin treatment at both doses (0.625 and 1.25 mg/kg) significantly (p<0.05) attenuated the increase in relative weight of kidneys compared to cisplatin alone treated rats (Figure 2D). Histopathological findings (Figure 3) of kidney tissues from vehicle control rats (Control) showed intact histo-morphology, whereas kidney tissues from cisplatin alone administered rats (Cis) revealed severe tubular degeneration and necrosis in the tubular epithelium (white arrow), infiltration of inflammatory cells (black arrow) and accumulation of homogenous eosinophilic casts (yellow arrow) in the lumen of the tubules. Kidney tissue sections from fisetin at low dose (0.625 mg/kg) with cisplatin treated group (Cis+Fis-0.625) showed decrease in tubular degeneration (white arrow), infiltration of inflammatory cells (black arrow) and accumulation of eosinophilic casts (yellow arrow) in tubular lumen when compared to cisplatin alone treated group of rats. However, animals treated with fisetin at 1.25 mg/kg along with cisplatin showed predominantly normal renal histology with occasional degenerative changes (white arrow) when compared to cisplatin alone treated rats (Cis+Fis-1.25).

### Effect of fisetin on cisplatin-induced changes in kidney tissues antioxidant and lipid peroxidation parameters

Table 1 depicts the activities and/or levels of various enzymatic (GR, GST, CAT, SOD and NQO1) and non-enzymatic (GSH and Vit C) antioxidants in the kidney tissues of all experimental groups. The activities of enzymatic and levels of non-enzymatic antioxidants were significantly (p<0.05: GSH, GR, NQO1 and Vit C; p<0.01: GST, CAT and SOD) decreased in kidney tissues of cisplatin alone administered rats. Fisetin administration at high dose (1.25 mg/kg) along with cisplatin significantly (p<0.05: GSH; p<0.01: GR, CAT and Vit C; p<0.001: GST, SOD and NQO1) restored the activities of these antioxidants to nearly normal. Though fisetin treatment at low dose (0.625 mg/kg) along with cisplatin was able to significantly (p<0.05: GSH; p<0.01: GST, SOD and NQO1) restore the activities and/or levels of GSH, GST, SOD and NQO1, the activities of GR, CAT and levels of vitamin C remains unaltered. Similarly, the level of TBARS, which measures the extent of lipid peroxidation was significantly (p<0.05) increased in cisplatin treated group of rats when compared to vehicle control rats. Treatment with fisetin at both the doses (0.625 and 1.25 mg/kg) significantly (p<0.05) decreased the level of TBARS when compared to cisplatin alone treated group of rats.

**Table 1:** Effect of fisetin on cisplatin-induced changes in renal antioxidants.

	Control	Fis	Cis	Cis+Fis-0.625 mg	Cis+Fis-1.25 mg
GSH levels	0.59±0.034	0.63±0.02	0.44±0.03$	0.67±0.05*	0.70±0.05*
GR activity	7.36±0.56	7.26±0.83	5.22±0.48$	7.10±0.31	8.49±0.95**
GST activity	15.98±0.79	16.46±1.33	7.42±1.47#	14.78±0.61**	18.69±2.04***
CAT activity	12.26±0.50	13.24±0.51	6.58±0.81#	7.87±0.38	11.10±0.93**
SOD activity	100.00±0.85	99.47±0.88	84.41±1.80#	96.4±1.02**	99.69±1.18***
NQO1 activity	127.81±5.97	132.38±4.20	89.62±18.22$	137.3±8.51**	152.86±4.49***
Vit C levels	0.82±0.05	1.37±0.13	0.38±0.08$	0.69±0.17	1.06±0.11**
TBARS levels	11.47±0.74	11.60±2.98	22.82±4.96$	10.79±2.46*	10.51±2.00*

All data were expressed as mean ± S.E.M. Where, Control, vehicle control (N = 7); Fis, fisetin alone treated group (N = 8); Cis, cisplatin alone treated group (N = 8); Cis+Fis-0.625 mg, fisetin (0.625 mg/kg) treated cisplatin-induced rats (N = 8); Cis+Fis-1.25 mg, fisetin (1.25 mg/kg) treated cisplatin-induced rats (N = 8); GSH, reduced glutathione (mg/g of tissue); GR, glutathione reductase (U/mg protein); GST, glutathione S-transferase (nmoles of CDNB conjugated/min/ml); CAT, catalase (U/mg protein); SOD, superoxide dismutase (% of control); NQO1, NAD (P) H: quinine oxidoreductase 1 (nmoles of DCIP reduced/min/mg protein); Vit C, vitamin C (mg/g of tissue); TBARS, thiobarbituric acid reactive substances (nmoles/g of tissue); CDNB, 1-chloro-2, 4-dinitrobenzene; DCIP, 2,6-dichlorophenolindophenol.

$ p<0.05,

# p<0.01 vs control,

*p<0.05,

**p<0.01,

***p<0.001 vs Cis control.

### Effect of fisetin on NADPH oxidase activity and mRNA expression of NOX2 and NOX4

**Figure 4:**
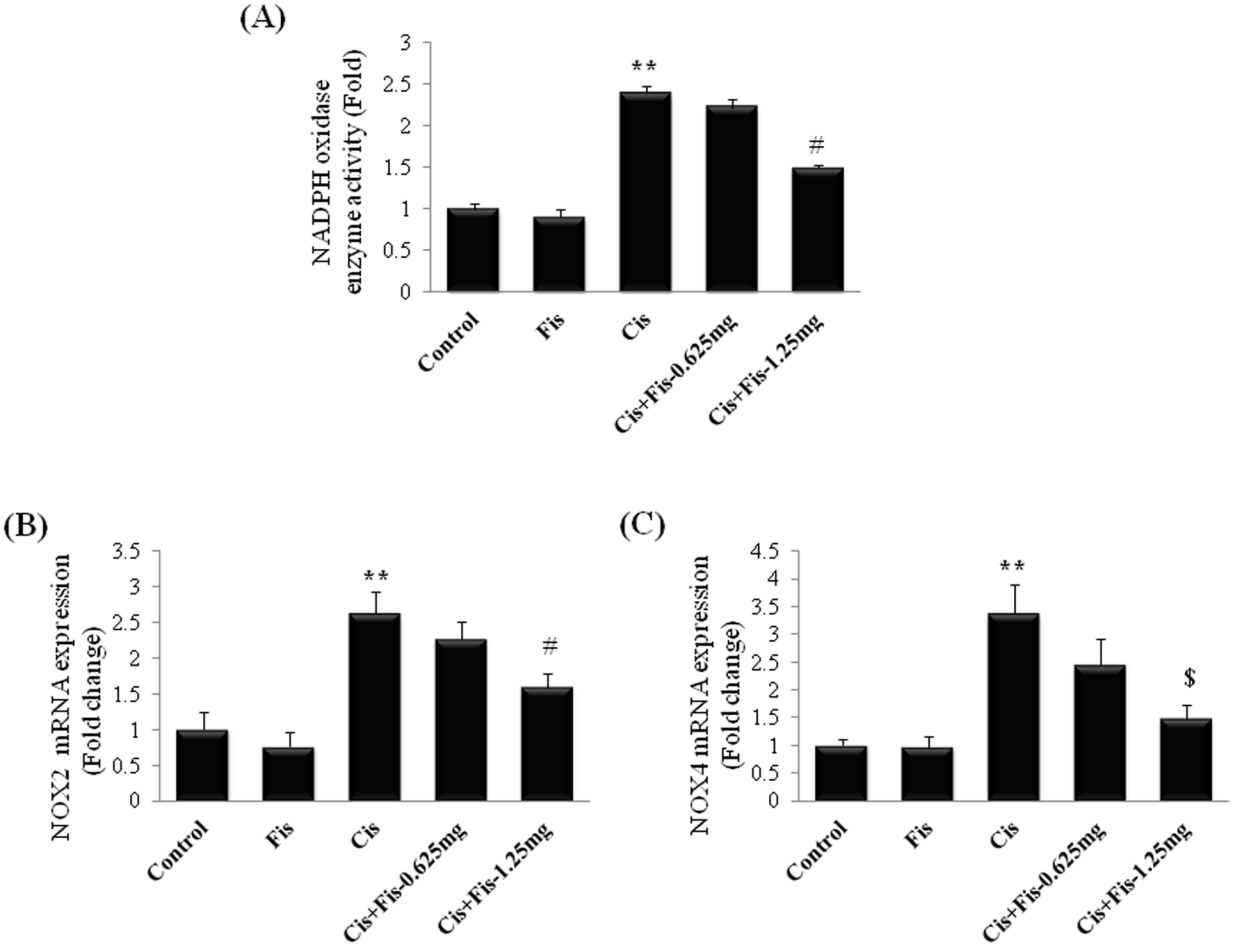
Effect of fisetin on cisplatin-induced changes in (A) NADPH oxidase enzyme activity and mRNA expressions of (B) NOX2/gp91phox and (C) NOX4/RENOX. The results were expressed as mean ± S.E.M of 4 animals in each group. Where, Control, vehicle control; Fis, fisetin control; Cis, cisplatin control; Cis+Fis-0.625 mg, cisplatin plus fisetin (0.625 mg/kg) treated groups; Cis+Fis-1.25 mg, cisplatin plus fisetin (1.25 mg/kg) treated groups. ^**^p<0.05 vs vehicle control group; ^#^p<0.05, ^$^p<0.01 vs cisplatin control group.

As shown in Figure 4, cisplatin administration significantly (2.4 fold) increased the activity of NADPH oxidase, whereas fisetin (1.25 mg/kg) treatment significantly (p<0.05) attenuated the cisplatin-induced increase in NADPH oxidase activity in kidney tissues. qPCR data revealed that the expressions of NOX2 and NOX4 were significantly (NOX2, 2.6 fold; NOX4, 3.4 fold) increased in cisplatin alone treated rats compared to vehicle control rats. Fisetin treatment at the high dose (1.25 mg/kg) significantly (NOX2, p<0.05; NOX4, p<0.01) attenuated the cisplatin-induced expressions of NOX2 and NOX4 in kidney tissues when compared to cisplatin alone treated rats. Rats treated with fisetin at low dose (0.625 mg/kg) along with cisplatin did not produce any significant (p>0.05) change in activity or mRNA expression of NOX compared to those of cisplatin alone treated rats.

### Effect of fisetin on cisplatin-induced changes in mitochondrial respiratory enzyme activities in kidney tissues

**Figure 5:**
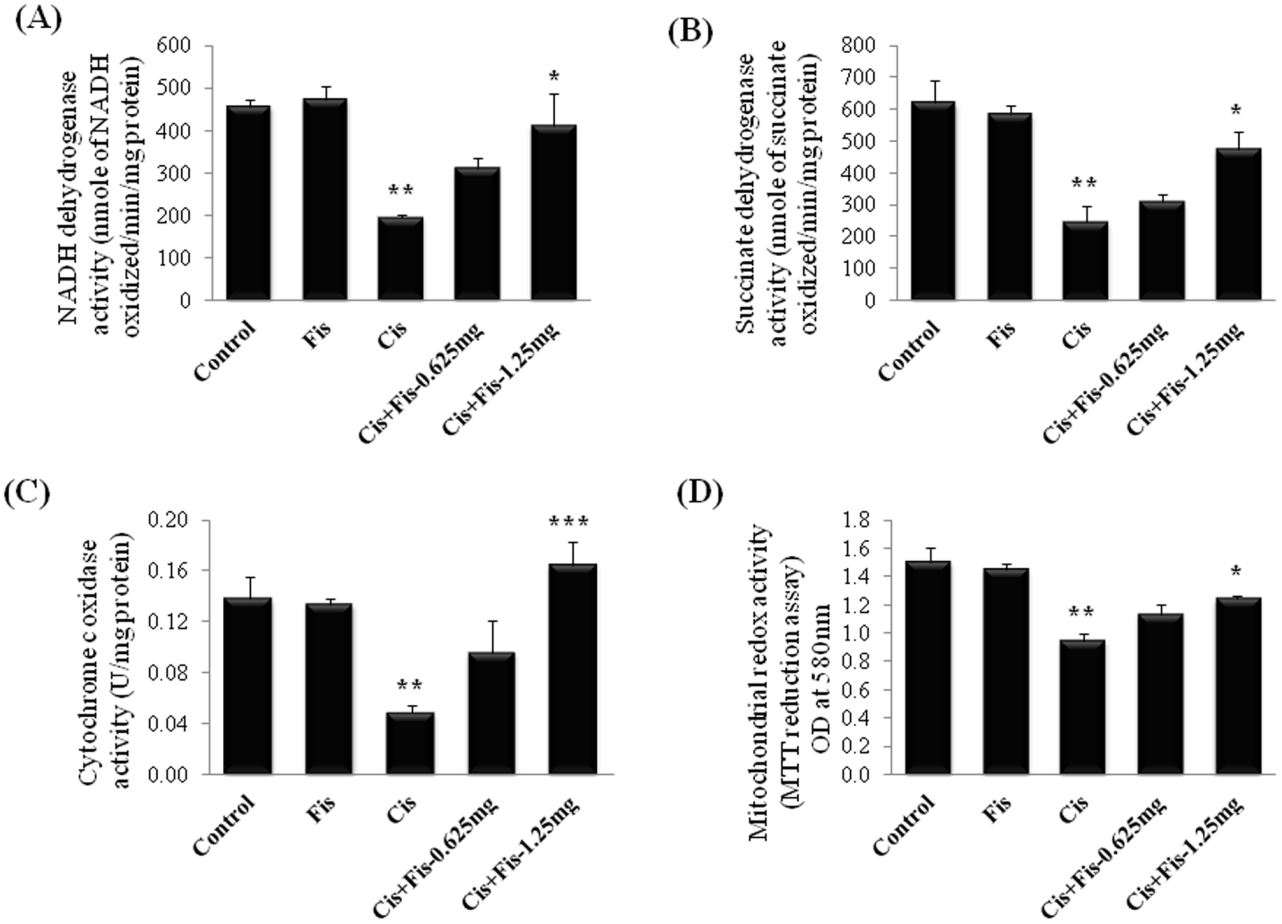
Effect of fisetin on cisplatin-induced changes in mitochondrial respiratory enzyme activities in kidney tissues. (**A**) NADH dehydrogenase, (**B**) Succinate dehydrogenase, (**C**) Cytochrome c oxidase and (**D**) Mitochondrial redox activities. The results were expressed as mean ± S.E.M of 6 animals in each group. Where, Control, vehicle control; Fis, fisetin control; Cis, cisplatin control; Cis+Fis-0.625 mg, cisplatin plus fisetin (0.625 mg/kg) treated groups; Cis+Fis-1.25 mg, cisplatin plus fisetin (1.25 mg/kg) treated groups. ^*^p<0.05 and ^***^p<0.001vs cisplatin control group; ^**^p<0.01 vs vehicle control group.

In order to investigate the effect of fisetin on mitochondrial function, mitochondrial respiratory enzymes such as NADH dehydrogenase, succinate dehydrogenase, cytochrome c oxidase and mitochondrial redox activity were evaluated in isolated mitochondrial fraction of kidney tissues of all experimental rats. As shown in Figure 5, the activities of NADH dehydrogenase (NDH) (Figure 5A), succinate dehydrogenase (SDH) (Figure 5B), cytochrome c oxidase (COX) (Figure 5C) and mitochondrial redox activities (Figure 5D) were significantly (p<0.01) decreased in cisplatin alone treated rats. Rats treated with fisetin at low dose (0.625 mg/kg) along with cisplatin did not produce any significant (p>0.05) change in these enzymes compared to those of cisplatin control group of rats. In contrast, the activities of these mitochondrial respiratory enzymes and mitochondrial redox activity were significantly (p<0.05 for NDH, SDH and redox activity; p<0.001 for COX) restored in high dose (1.25 mg/kg) fisetin treatment group when compared to cisplatin alone treated group of rats.

### Effect of fisetin on cisplatin-induced changes in mitochondrial antioxidant and lipid peroxidation parameters in kidney tissues

Table 2 represents the effect of fisetin on the cisplatin-induced changes in the mitochondrial antioxidants in the kidney tissues of all experimental rats. Cisplatin administration caused a significant (p<0.05: GR, GST and CAT; p<0.01: GSH and SOD) reduction in the levels of mitochondrial antioxidants such as GSH, GR, GST, SOD and CAT in kidney tissues. Fisetin treatment at high dose (1.25 mg/kg) along with cisplatin significantly (p<0.05: GR, GST and CAT; p<0.01: GSH and SOD) restored the level of these enzymes when compared to cisplatin control rats. Though fisetin treatment at low dose (0.625 mg/kg) restored the levels of mitochondrial activities of SOD and CAT, the levels of GSH, GR and GST remain unaltered when compared to those of cisplatin alone treated rats. In addition, to assess the mitochondrial lipid peroxidation status, TBARS content was estimated. TBARS content in cisplatin alone treated rats was significantly (p<0.01) increased when compared to those of vehicle control rats. Fisetin treatment at both the doses (0.625 and 1.25 mg/kg) significantly (p<0.05: for 0.625 mg/kg fisetin dose; p<0.01: for 1.25 mg/kg fisetin dose) decreased the TBARS level when compared to cisplatin alone treated group of rats.

**Table 2:** Effect of fisetin on cisplatin-induced changes in renal mitochondrial antioxidants.

	Control	Fis	Cis	Cis+Fis-0.625 mg	Cis+Fis-1.25 mg
GSH levels	45.72±0.98	45.33±1.99	34.50±1.15#	38.88±1.22	45.10±1.66**
GR activity	3.20±0.17	3.32±0.23	2.34±0.06$	2.70±0.21	3.48±0.28*
GST activity	126.72±8.56	127.40±5.48	105.92±3.45$	123.67±5.36	142.3±3.84*
CAT activity	3.59±0.19	3.74±0.30	2.55±0.19$	3.40±0.25*	3.51±0.14*
SOD activity	100.00±0.38	99.86±1.18	88.61±0.86#	95.88±1.32*	98.19±0.52**
TBARS levels	5.92±0.54	5.95±0.32	10.68±0.87#	7.04±0.69*	5.57±0.26**

All data were expressed as mean± S.E.M. Where, Control, vehicle control (N = 7); Fis, fisetin alone treated group (N = 8); Cis, cisplatin alone treated group (N = 8); Cis+Fis-0.625 mg, fisetin (0.625 mg/kg) treated cisplatin-induced rats (N = 8); Cis+Fis-1.25 mg, fisetin (1.25 mg/kg) treated cisplatin-induced rats (N = 8); GSH, reduced glutathione (µg/g of tissue); GR, glutathione reductase (U/mg protein); GST, glutathione S-transferase (nmoles of CDNB conjugated/min/ml); CAT, catalase (U/mg protein); SOD, superoxide dismutase (% of control); TBARS, thiobarbituric acid reactive substances (nmoles/g of tissue); CDNB, 1-chloro-2, 4-dinitrobenzene.

$ p<0.05,

# p<0.01 vs control,

*p<0.05,

**p<0.01 vs Cis control.

### Effect of fisetin on cisplatin-induced changes in inflammatory markers

**Figure 6:**
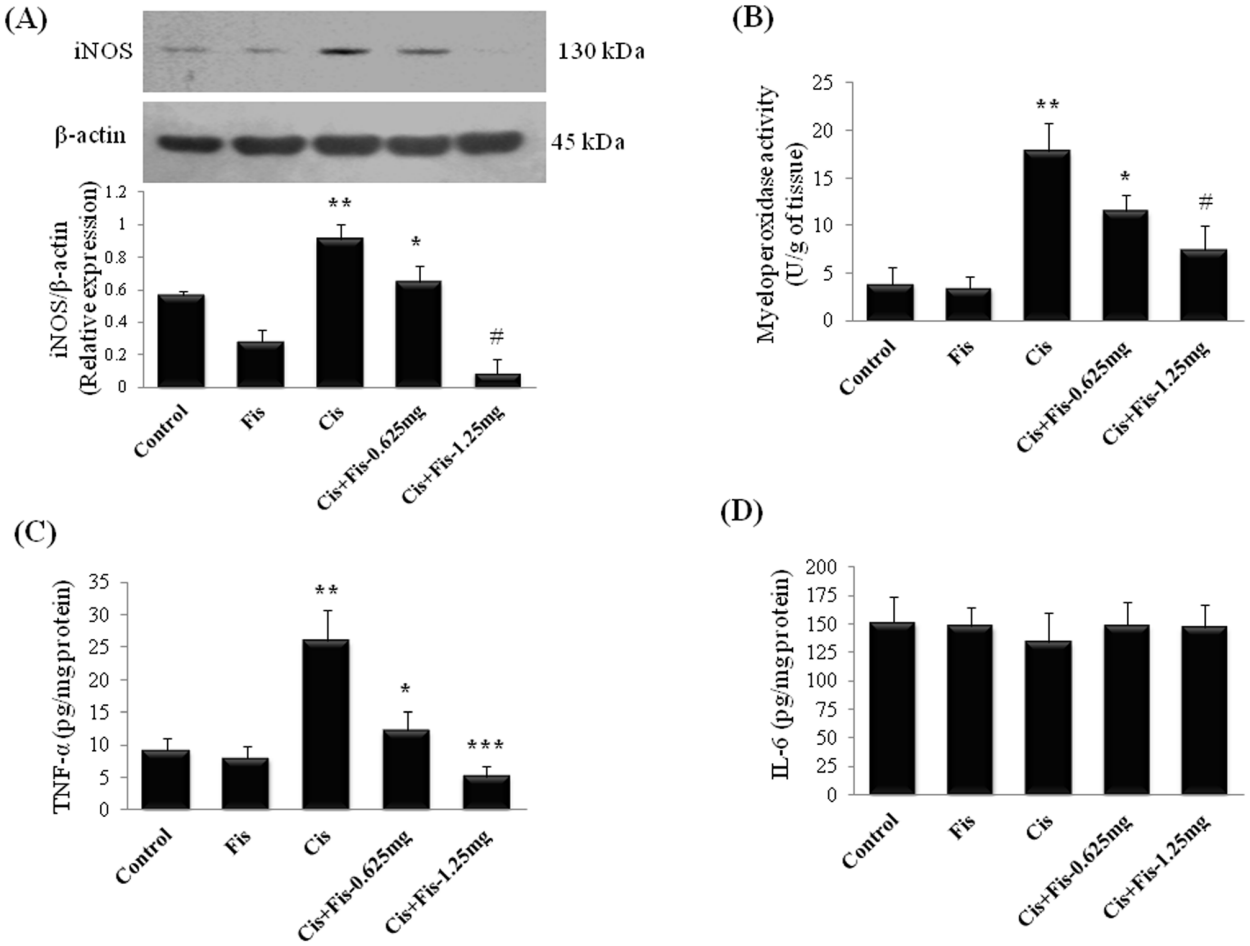
Effect of fisetin on cisplatin-induced renal inflammation. (**A**) Effect of fisetin on cisplatin-induced changes in protein expression of inducible nitric oxide (iNOS) in kidney tissues of control and treated groups. Renal tissue total protein extract was prepared and analyzed for expression level of iNOS by western blotting using specific antibodies. β-actin was used as loading control for equal loading of proteins. Protein expression was studied for three independent experiments and representative blots are shown. Quantitative densitometry was performed for each blot. Fisetin at both the doses (0.625 and 1.25 mg/kg) significantly decreased the protein expression of iNOS compared to cisplatin control. Effect of fisetin on cisplatin-induced changes in (**B**) Myeloperoxidase activity, as an index for neutrophil infiltration; changes in proinflammatory cytokine levels (**C**) Tumor necrosis factor-α (TNF-α) and (**D**) Interleukin-6 (IL-6). The results were expressed as mean ± S.E.M of 6 animals in each group. Where, Control, vehicle control; Fis, fisetin control; Cis, cisplatin control; Cis+Fis-0.625 mg, cisplatin plus fisetin (0.625 mg/kg) treated groups; Cis+Fis-1.25 mg, cisplatin plus fisetin (1.25 mg/kg) treated groups. ^*^p<0.05, ^#^p<0.01 and ^***^p<0.001vs cisplatin control group; ^**^p<0.01 vs vehicle control group.

In order to evaluate whether fisetin was able to attenuate the cisplatin evoked renal inflammation, we analyzed the pro-inflammatory cytokine levels i.e. TNF-α, IL-6, myeloperoxidase activity and renal protein expression of inducible nitric oxide synthase (iNOS) in kidney tissues. As shown in Figure 6, the protein expression of iNOS (Figure 6A); the myeloperoxidase (MPO) activity (Figure 6B), a marker for leukocytes/macrophage infiltration; TNF-α (Figure 6C), a pro-inflammatory cytokine were significantly (p<0.01) increased in kidney tissues of cisplatin alone treated rats when compared to those of vehicle control rats. Fisetin administration significantly attenuated the cisplatin-induced increase in the renal protein expression of iNOS, the activities of MPO and pro-inflammatory cytokines, TNF-α when compared to cisplatin alone treated rats. The IL-6 levels remain unchanged in fisetin plus cisplatin treated rats when compared to vehicle as well as cisplatin control group of rats (Figure 6D). Fisetin alone did not produce any significant change in these inflammatory markers when compared to vehicle control group of rats

**Figure 7:**
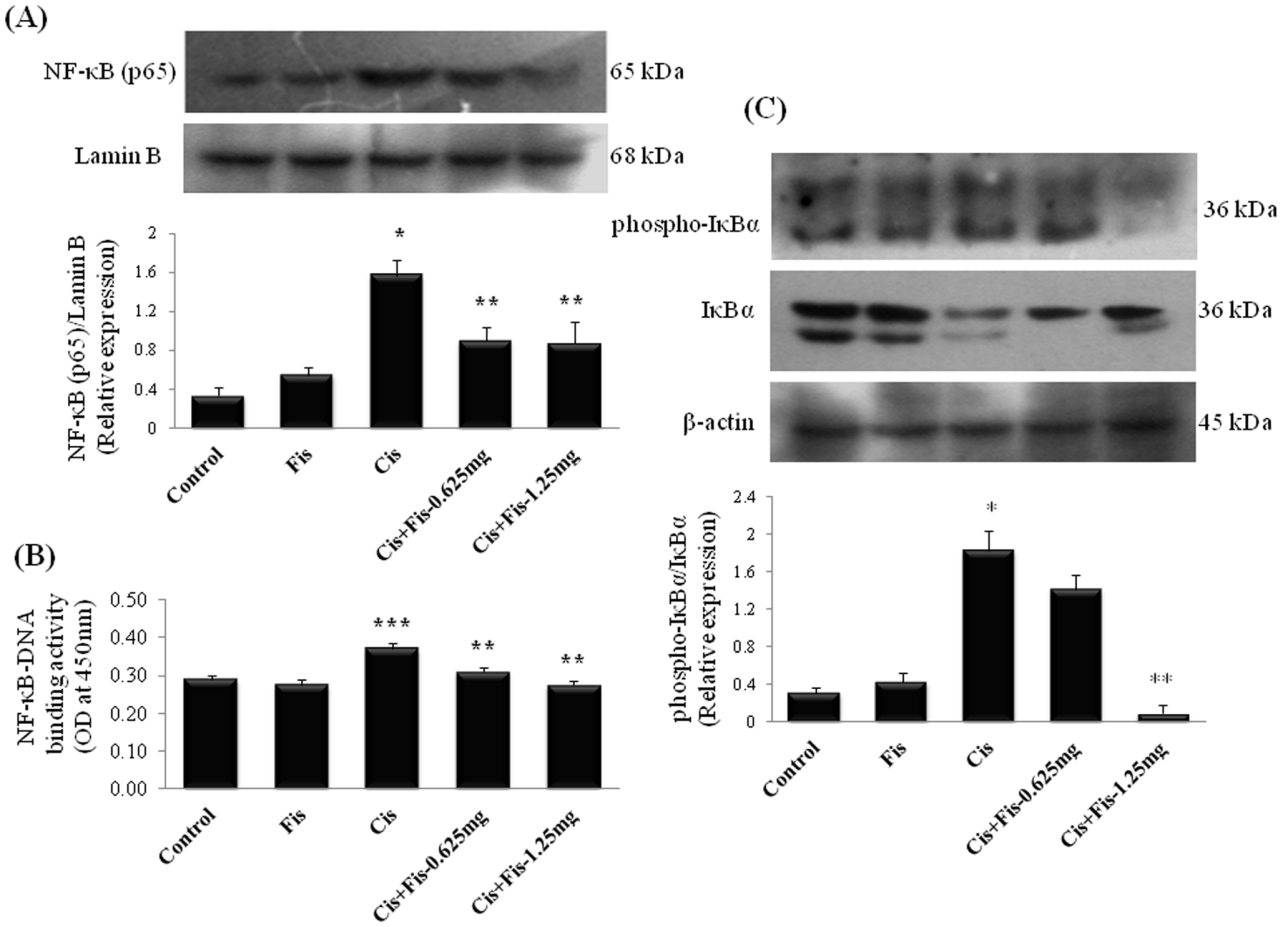
Effect of fisetin on cisplatin-induced NF-κB (p65) protein activation and IκBα phosphorylation. (**A**) Effect of fisetin on cisplatin-induced changes in kidney tissue nuclear protein expression of NF-κB (p65) and (**B**) DNA binding activity of NF-κB (p65). Kidney tissue nuclear protein extract was used for estimation of NF-κB (p65) - DNA binding activity using NF-κB (p^65^) transcription factor ELISA assay kit (Cayman Chemical Company, Ann Arbor, MI). The results were expressed as mean ± S.E.M of 6 animals in each group. (**C**) Kidney tissue cytoplasmic protein expression of phospho-IκBα and IκBα. β-actin and Lamin-B were used as loading control for equal loading of cytoplasmic and nuclear proteins respectively. Protein expressions were studied for three independent experiments and representative blots are shown. Quantitative densitometry was performed for each blot. Where, Control, vehicle control; Fis, fisetin control; Cis, cisplatin control; Cis+Fis-0.625 mg, cisplatin plus fisetin (0.625 mg/kg) treated groups; Cis+Fis-1.25 mg, cisplatin plus fisetin (1.25 mg/kg) treated groups. ^*^p<0.05, ^***^p<0.001vs vehicle control group; ^**^p<0.01 vs cisplatin control group.

To further investigate the mechanisms underlying the beneficial effects of fisetin on cisplatin induced renal inflammation, we analyzed the protein expression and activation of NF-κB (p65) in kidney tissues. Results from immunoblot analysis showed the amount of NF-κB (p65) in the nuclear protein fraction of kidney tissues from cisplatin alone administered rats were significantly (p<0.05) increased when compared to vehicle control rats (Figure 7A). To further clarify the involvement of NF-κB, NF-κB (p65) transcription assay was performed. As expected the DNA binding activity of NF-κB (p65) was significantly (p<0.001) increased in cisplatin alone treated rats (Figure 7B). Fisetin administration at both the doses (0.625 and 1.25 mg/kg) along with cisplatin significantly (p<0.01) decreased the amount of NF-κB (p65) and NF-κB (p65)-DNA binding activity when compared to cisplatin alone treated rats. Additionally, the amount of IκBα protein was down regulated with concomitant increase in phospho-IκBα in cisplatin alone administered rats (Figure 7C). Fisetin administration at higher dose (1.25 mg/kg) decreased the phosphorylation of IκBα protein and enhanced the IκBα protein level in kidney tissues when compared to cisplatin alone treated rats.

### Effect of fisetin on cisplatin-induced changes in renal apoptosis related proteins

**Figure 8:**
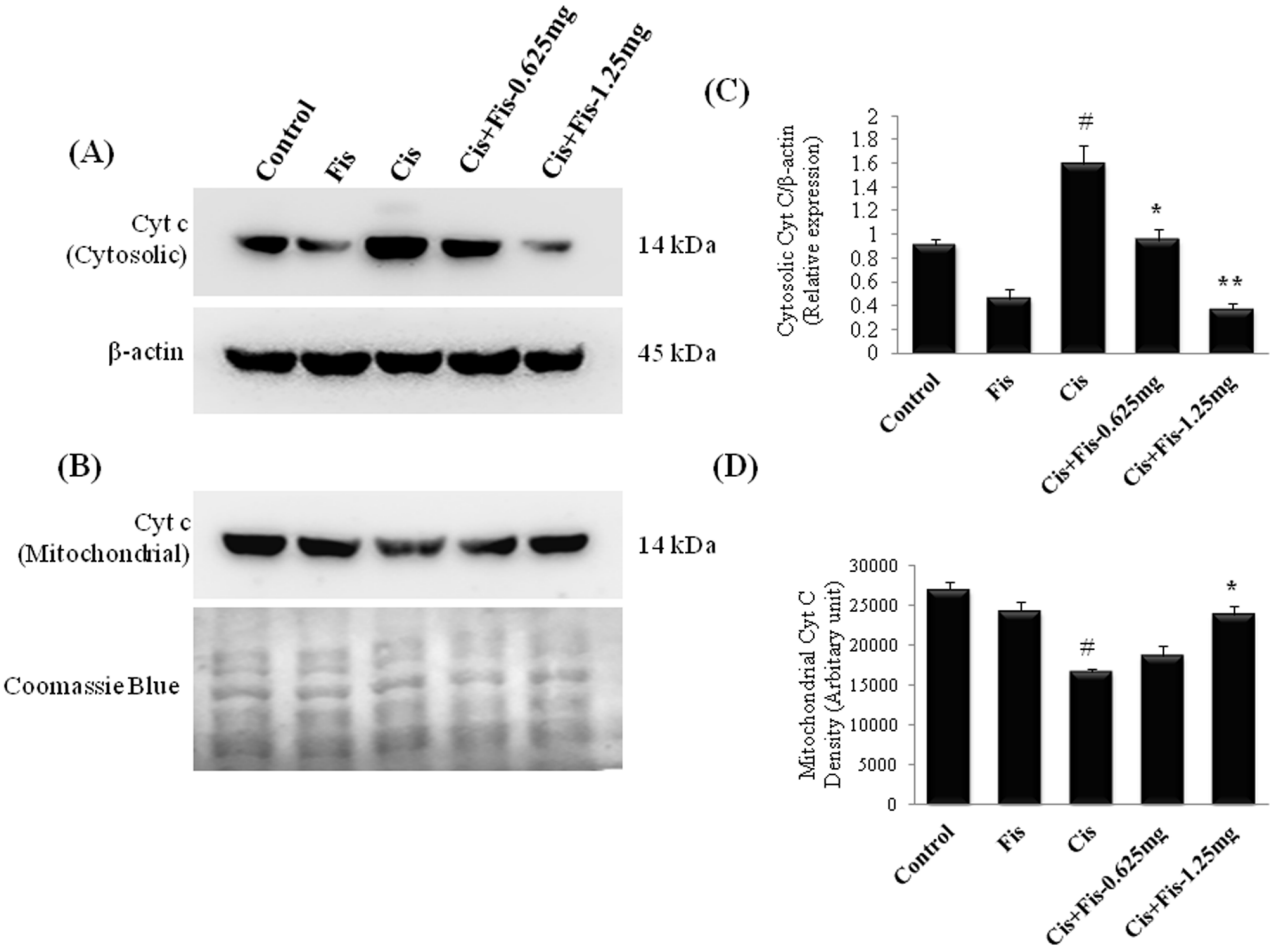
Effect of fisetin on cisplatin-induced cytosolic translocation of cytochrome c. Fisetin at both the doses (0.625 and 1.25 mg/kg) significantly attenuated the cytosolic translocation of cytochrome c protein (**A**) from the mitochondria (**B**) compared to cisplatin alone treated group. β-actin and Coomassie blue were used as loading control for equal loading of cytosolic and mitochondrial proteins respectively. Protein expressions were studied for three independent experiments and representative blots are shown. Quantitative densitometry was performed for each blot. (**C**) Densitometric analysis of cytosolic cytochrome c. (**D**) Densitometric analysis of mitochondrial cytochrome c. Where, Control, vehicle control; Fis, fisetin control; Cis, cisplatin control; Cis+Fis-0.625 mg, cisplatin plus fisetin (0.625 mg/kg) treated groups; Cis+Fis-1.25 mg, cisplatin plus fisetin (1.25 mg/kg) treated groups. ^#^p<0.05 vs vehicle control group; ^*^p<0.05 and ^**^p<0.01 vs cisplatin control group.

**Figure 9:**
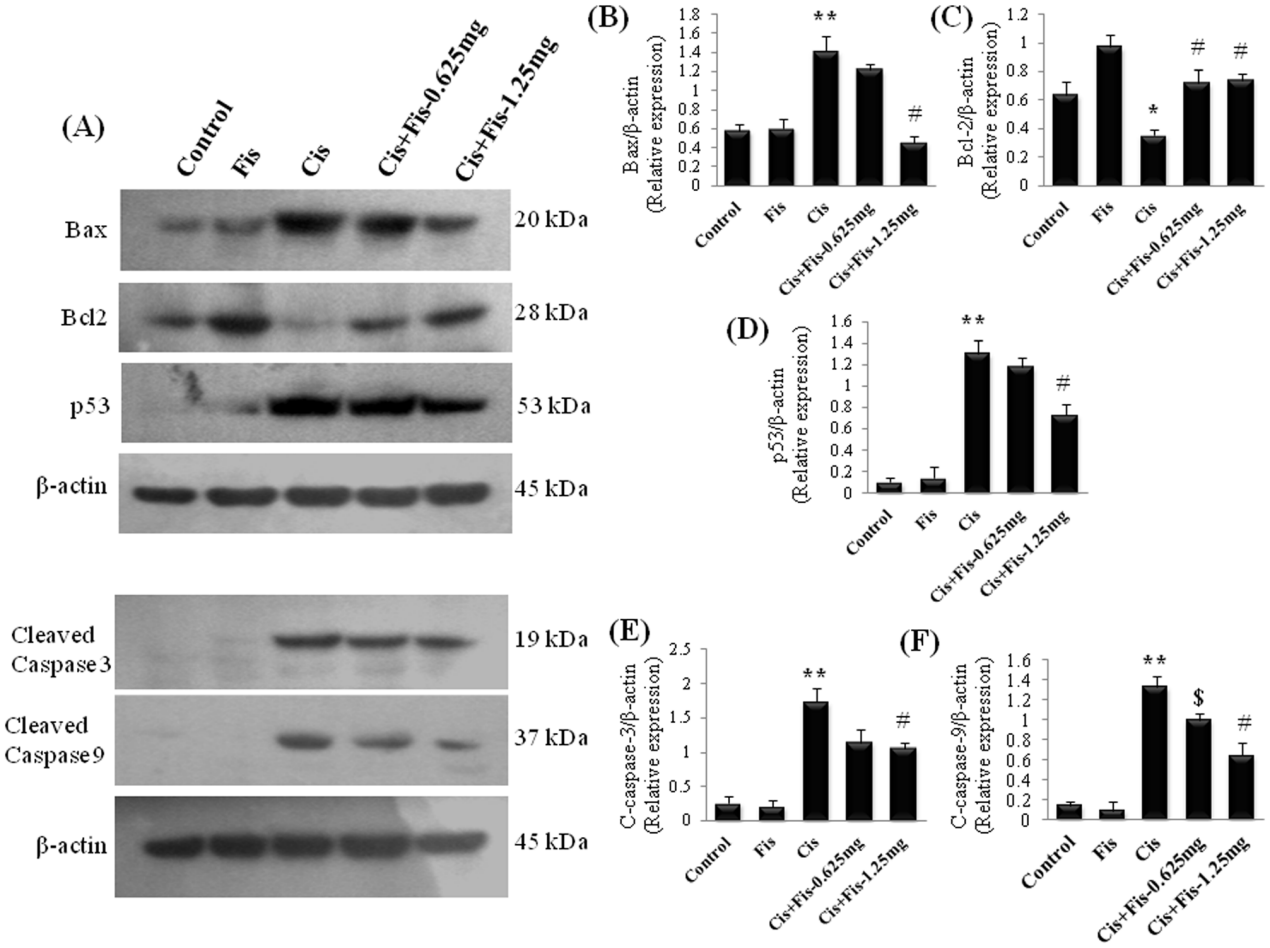
Western blot analyses of apoptosis related proteins in kidney tissues. Kidney tissue total protein extract was prepared and aliquots of 40 µg of protein extracts from different experimental groups were separated by SDS-PAGE and transferred to PVDF membrane. Western blot analyses were performed for Bax, Bcl2, p53, cleaved caspase-3 and cleaved caspase-9 using specific antibodies. β-actin was used as loading control for equal loading of proteins. Quantitative densitometry was performed for each blot. Protein expressions were studied for three independent experiments and representative blots are shown. Where, Control, vehicle control; Fis, fisetin control; Cis, cisplatin control; Cis+Fis-0.625 mg, cisplatin plus fisetin (0.625 mg/kg) treated groups; Cis+Fis-1.25 mg, cisplatin plus fisetin (1.25 mg/kg) treated groups. ^*^p<0.05 and ^**^p<0.01vs vehicle control group; ^$^p<0.05 and ^#^p<0.01 vs cisplatin control group.

In order to elucidate the effect of fisetin on cisplatin-induced renal cell apoptosis, we analysed the various apoptosis related proteins such as cytochrome c, Bax, Bcl-2, cleaved caspase-3, cleaved caspase-9 and p53 in kidney tissues. Cisplatin significantly (p<0.05) enhanced the cytosolic translocation of cytochrome c from mitochondrial fraction when compared to vehicle control rats (Figure 8). Furthermore, the protein expressions of Bax, p53, cleaved caspase-3 and cleaved caspase-9 were significantly (p<0.01) increased and the protein expression of Bcl-2 was significantly (p<0.05) decreased in cisplatin alone administered rats when compared to those of vehicle control rats (Figure 9). Fisetin treatment at higher dose (1.25 mg/kg) along with cisplatin significantly attenuated the cytosolic translocation of cytochrome c, protein expression of Bax, p53, cleaved caspase-3 and cleaved caspase-9 and increased the expression of Bcl-2 when compared to those of cisplatin alone treated rats.

### Effect of fisetin on platinum accumulation in kidney tissues

**Figure 10:**
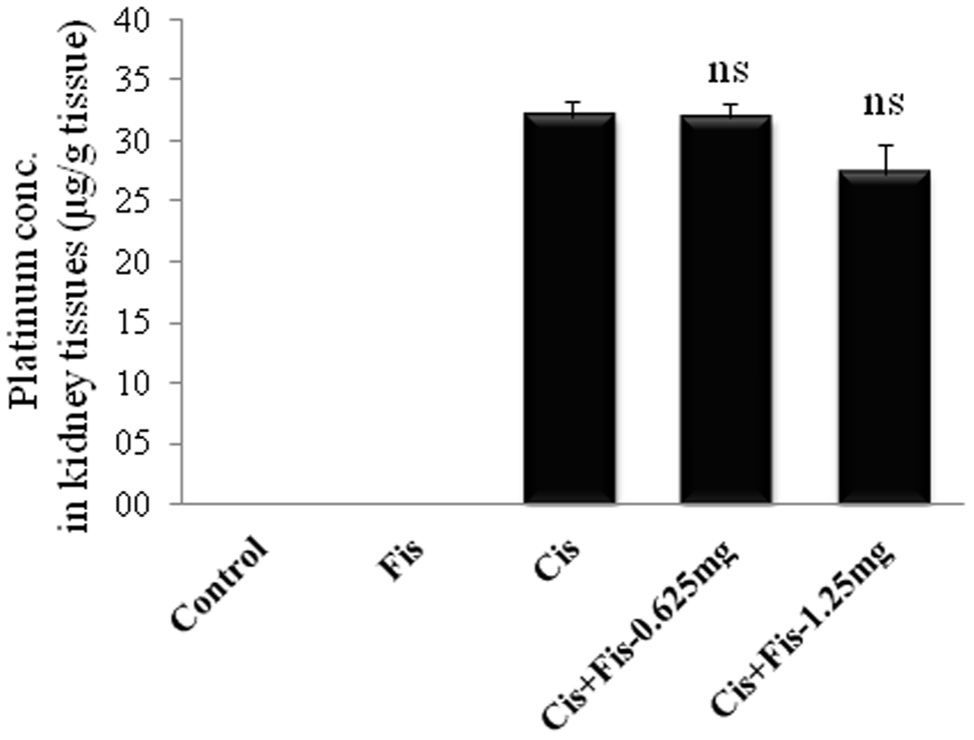
Effect of fisetin on platinum accumulation in kidney tissues. Platinum concentration in kidney tissues was estimated by using Inductively Coupled Plasma Mass Spectrometry (ICP-MS) and was expressed as µg/g of kidney tissues. Data revealed that fisetin treatment did not alter the cisplatin uptake in kidney tissues. The results were expressed as mean ± S.E.M of 6 animals in each group. Where, Control, vehicle control; Fis, fisetin control; Cis, cisplatin control; Cis+Fis-0.625 mg, cisplatin plus fisetin (0.625 mg/kg) treated groups; Cis+Fis-1.25 mg, cisplatin plus fisetin (1.25 mg/kg) treated groups. ns: non-significant.

To assess the effect of fisetin on platinum accumulation in renal tissue, we next analyzed the platinum concentration in kidney tissues of all experimental groups. As shown in Figure 10, we have not observed any significant change in platinum concentration between cisplatin alone and cisplatin plus fisetin treated (0.625 and 1.25 mg/kg) group of rats.

## Discussion

Nephrotoxicity is a frequent devastating adverse effect of cisplatin chemotherapy. The unique pharmacological profile of fisetin has attracted considerable attention in the field of cancer research. In a previous study, it has been demonstrated that combination of fisetin along with cisplatin exhibited four times more anticancer potential than individual treatment group [20]. In the present study, we demonstrated that fisetin treatment along with cisplatin largely reduced the nephrotoxicity, a clinical-utility limiting side effect of the cisplatin chemotherapy, by employing rat model. The findings of the present study revealed that fisetin treatment reduced the cisplatin-induced renal and mitochondrial oxidative stress, restored mitochondrial respiratory enzyme activities and attenuated expressions of apoptosis and inflammation related proteins, thus forming the molecular basis for protective mechanism of fisetin against cisplatin-induced nephrotoxicity.

In agreement with previous reports, we found that a single intraperitoneal injection of cisplatin (5 mg/kg) induced marked elevation of BUN and creatinine (renal function biomarkers) in serum (Figure 2) and also demonstrated histopathological damage with tubular degeneration, tubular necrosis and infiltration of inflammatory cells in kidneys (Figure 3). Although the precise molecular mechanism of cisplatin-induced nephrotoxicity is complex and remains uncertain, induction of oxidative damage, apoptosis/necrosis of renal tubular cells and activation of inflammatory pathways have been demonstrated in kidneys of cisplatin treated animals [37]. Most of the literatures, including our earlier studies, have revealed that cisplatin induces free radicals and produces oxidative damage and lipid peroxidation in kidney tissues [38], [39]. Cisplatin generates highly reactive free radicals such as superoxide and hydroxyl radicals which can directly interact and modify many subcellular components including DNA, proteins, lipids and other macromolecules and eventually causes cell death [40]. Cisplatin induced ROS generation in renal tubular cells activates NF-κB and thus is responsible for augmentation of iNOS and pro-inflammatory cytokine, chemokines and adhesion molecules expressions along with infiltration of inflammatory cells in renal tubules [3], [10], [41]. The importance of NOX2/gp91phox and NOX4/RENOX as a source of ROS generation, in particular superoxide, in the kidneys under cisplatin insult and other pathological conditions is also documented [3], [10]. The present study also revealed oxidative damage in kidney tissues of cisplatin treated group of rats. Fisetin has been reported to possess anti-inflammatory properties as well as good antioxidant property with a trolox equivalent antioxidant capacity (TEAC) value of ∼3. The NF-E2-related factor 2 (Nrf2) dependent inductions of phase II detoxifying enzymes of fisetin in HT22 cells, retinal ganglion cells, and primary cortical neurons has also been reported [12]. In the present study, cisplatin-induced increase in renal NADPH oxidase activities, expressions of NOX2 and NOX4 and thereby cellular ROS production was attenuated by fisetin treatment (Figure 4). Fisetin treatment also restored the renal antioxidants including the level and/or activity of GSH, CAT, GR, GST, NQO1 and SOD (Table 1). These findings strengthen the hypothesis that renoprotective effect of fisetin could be attributed to its free radical scavenging and strong antioxidant properties.

A growing body of evidence suggests the role of inflammation and iNOS-mediated increased nitrosative stress in cisplatin-induced kidney toxicity [3], [42]. A significant elevation of nuclear translocation (Figure 7A) and DNA binding activity of NF-κB (p65) (Figure 7B), increased iNOS expression (Figure 6A), myeloperoxidase activity (Figure 6B) and concentration of TNF-α (Figure 6C) in the kidneys of cisplatin administered rats was observed in the present study. Our results revealed that fisetin treatment effectively scavenged the cisplatin-induced ROS and suppressed NF-κB activation and subsequent NF-κB mediated inflammatory protein expression in kidney tissues. The findings of the present study also corroborates with earlier study in which fisetin attenuated NF-κB (p65) nuclear translocation and DNA-binding activity and attenuates allergic airway inflammation in mice [16]. Under physiological condition, NF-κB is sequestered in cytoplasm by IκBα subunit, however, on exposure to ROS, sequestration complex breaks down and IκBα is phosphorylated at serine residues by IKKs, allows NF-κB to translocate into the nucleus to promote transcription of inflammatory genes [43]. In the present study, we observed a significant increase in phosphorylated IκBα protein and decrease in intact IκBα protein in cisplatin treated rats when compared to those of vehicle control rats (Figure 7C). Fisetin treatment along with cisplatin preserved IκBα degradation, attenuated IκBα phosphorylation and subsequent NF- κB nuclear translocation. A number of studies reported that cisplatin administration caused a significant elevation of IL-6 levels in kidneys [44]. Contrary to this, we did not find any significant change in IL-6 levels in kidney tissues of cisplatin treated rats (Figure 6D). Studies have revealed dual role of IL-6 in terms of pro-inflammatory and anti-inflammatory response. Interleukin-6 is known to alleviates reactive oxygen species generation through heme oxygenase-1 (HO-1) induction and protecting renal tissues from cisplatin-induced toxicity [5]. At the same time, IL-6 also acts as downstream mediator in TNF-α/NF-κB signalling pathway. However, further studies are advocated to confirm this.

In physiological conditions, mitochondria continuously generate small quantity of superoxide free radicals by converting 1–2% of consumed oxygen and act as important source of ROS. Oxidative damage of mitochondria alters the mitochondrial redox function and respiratory chain enzymes, leading to over production of free radicals and cellular dysfunction [45]. Generation of free radicals and mitochondrial oxidative stress-induced dysfunction have been implicated as early events in the pathogenesis of cisplatin-induced nephrotoxicity [9], [10], [40], [46]. It has been reported that endogenous free radical scavengers such as vitamin C and E, glutathione, ubiquinol, superoxide dismutase and glutathione peroxidase protect mitochondria from cisplatin-induced oxidative damage [45]. Recently, Mukhopadhyay et al. [10] demonstrated that a single systemic dose of mitochondrial targeted antioxidants, MitoQ and Mito-CP, that deliver superoxide dismutase mimetics preferentially in to mitochondria, significantly prevented cisplatin-induced renal dysfunction in mice. We found that fisetin treatment (1.25 mg/kg) significantly restored the cisplatin-induced decrease in mitochondrial respiratory chain enzyme activities such as NADH dehydrogenase (Figure 5A), succinate dehydrogenase (Figure 5B) and cytochrome c oxidase (Figure 5C). The decrease in mitochondrial redox activity which is a measure of functional intact mitochondria was also significantly increased in fisetin (1.25 mg/kg) treated cisplatin challenged rats (Figure 5D). Additionally, mitochondrial content of GSH, GST, GR, SOD and CAT activities were significantly restored and mitochondrial content of TBARS was significantly decreased in fisetin (1.25 mg/kg) treated cisplatin challenged group of rats (Table 2). These data indicates fisetin ameliorated mitochondrial oxidative damage and associated dysfunction and exhibited renoprotective effect against cisplatin-induced nephrotoxicity.

Apoptosis is a major cause of cisplatin-induced nephrotoxicity [6], [47]. To elucidate the nephroprotective mechanism of fisetin on cisplatin-induced tubular cell death, we investigated the expression of various apoptosis related proteins in kidney tissues. Our result revealed that fisetin treatment decreased the cisplatin-induced cytosolic translocation of cytochrome c (Figure 8) and protein expression of Bax and significantly increased the anti-apoptotic protein, Bcl-2 in kidney tissues (Figure 9). Additionally, the amount of cleaved caspase-9 and cleaved caspase-3 were significantly decreased in fisetin treated cisplatin-induced rats. These findings in our study indicate that fisetin may attenuate the cisplatin-induced tubular cell apoptosis through modulation of intrinsic mitochondrial apoptosis pathway. In addition to this, the p53 protein expression was also significantly increased in cisplatin control rats (Figure 9). It has been reported that pro-apoptotic role of p53 is predominant in cisplatin nephrotoxicity, inhibition of which significantly reduces tubular cell apoptosis [6]. Reports also suggest that the cisplatin-induced renal oxidative stress may activate cisplatin-induced p53 activation. Thus, in the present study, we believe that fisetin effectively scavenged cisplatin-induced ROS generation directly and/or indirectly through antioxidant mechanism and attenuated renal p53 expression and subsequent renal tubular cell death. Additionally, ICP-MS data revealed that fisetin did not alter the renal uptake of cisplatin (Figure 10). Hence, we suggest, fisetin by virtue of its free radical scavenging properties attenuates the cisplatin-induced nephrotoxicity in rats.

In conclusion, the results of the present study clearly demonstrated the renoprotective effect of fisetin against cisplatin-induced acute renal injury in rats. The mechanisms underlying the renoprotective effect of fisetin could be by reducing oxidative stress, restoring mitochondrial respiratory enzyme activities and suppressing apoptosis in renal tissues. In addition, the mechanism of this renoprotective effect may also involve inhibition of NF-κB activation and attenuation of subsequent pro-inflammatory mediators release in kidney tissues. However, further studies are needed to explore the additional mechanisms responsible for renoprotective effect of fisetin and to establish its feasible use in clinical setup as an adjunct candidate to cisplatin therapy.

## Supporting Information

Figure S1 **Effect of fisetin itself on serum cardiac injury biomarkers and heart histology.** Intraperitoneal administration of fisetin at two different doses i.e. 1.25 mg/kg and 2.5 mg/kg body weight for 7 consecutive days showing normal serum CK-MB (creatine kinase-MB isoenzyme) and LDH (lactate dehydrogenase) levels compared to vehicle control rats. Histopathological examination of heart tissue (X200 magnification, scale bar: 50 µm) from vehicle control (Control), fisetin at 1.25 mg/kg (Fis-1.25) and fisetin at 2.5 mg/kg (Fis-2.5) treated rats showing apparently normal histo-morphology.
(TIF)

Figure S2 **Effect of fisetin itself on serum liver injury biomarkers and liver histology.** Intraperitoneal administration of fisetin at two different doses i.e. 1.25 mg/kg and 2.5 mg/kg body weight for 7 consecutive days showing normal serum SGOT (serum glutamic oxaloacetic transaminase) and SGPT (serum glutamic pyruvic transaminase) levels compared to vehicle control rats. Histopathological examination of liver tissue (X200 magnification, scale bar: 50 µm) from vehicle control (Control), fisetin at 1.25 mg/kg (Fis-1.25) and fisetin at 2.5 mg/kg (Fis-2.5) treated rats showing apparently normal histo-morphology.
(TIF)
