# Supplementary figures and images for: Ameliorative Effect of Fisetin on Cisplatin-Induced Nephrotoxicity in Rats via Modulation of NF-κB Activation and Antioxidant Defence
Source: PLoS One. 2014 Sep 3;9(9):e105070. doi: 10.1371/journal.pone.0105070 (PMC4153571; doi:10.1371/journal.pone.0105070)

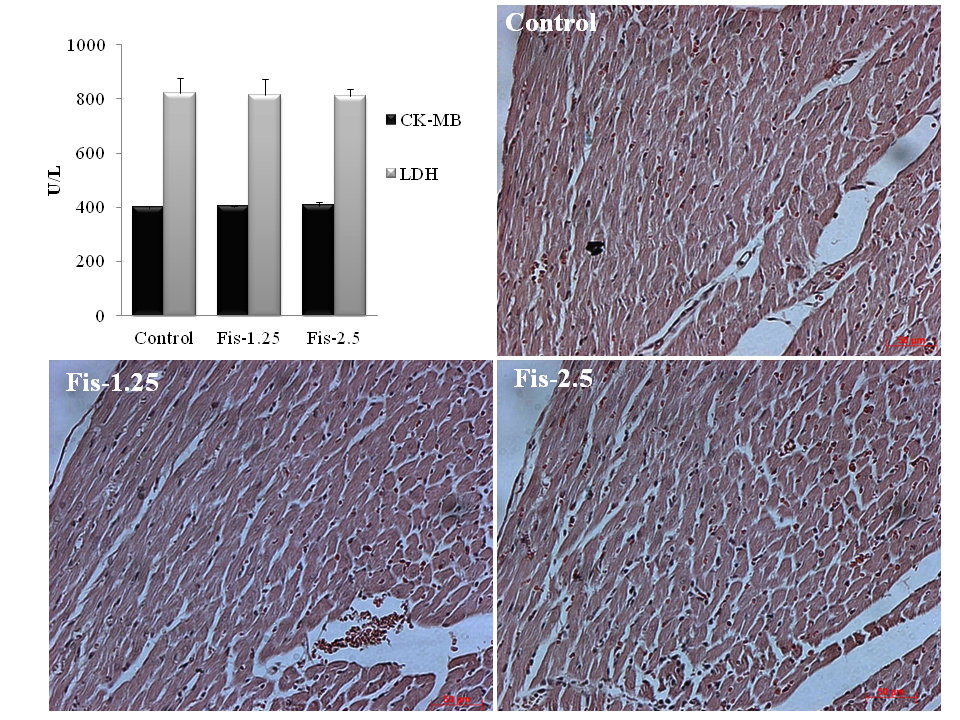

Supplement: Figure S1 — Effect of fisetin itself on serum cardiac injury biomarkers and heart histology. Intraperitoneal administration of fisetin at two different doses i.e. 1.25 mg/kg and 2.5 mg/kg body weight for 7 consecutive days showing normal serum CK-MB (creatine kinase-MB isoenzyme) and LDH (lactate dehydrogenase) levels compared to vehicle control rats. Histopathological examination of heart tissue (X200 magnification, scale bar: 50 µm) from vehicle control (Control), fisetin at 1.25 mg/kg (Fis-1.25) and fisetin at 2.5 mg/kg (Fis-2.5) treated rats showing apparently normal histo-morphology. (TIF) [file pone.0105070.s001.tif]

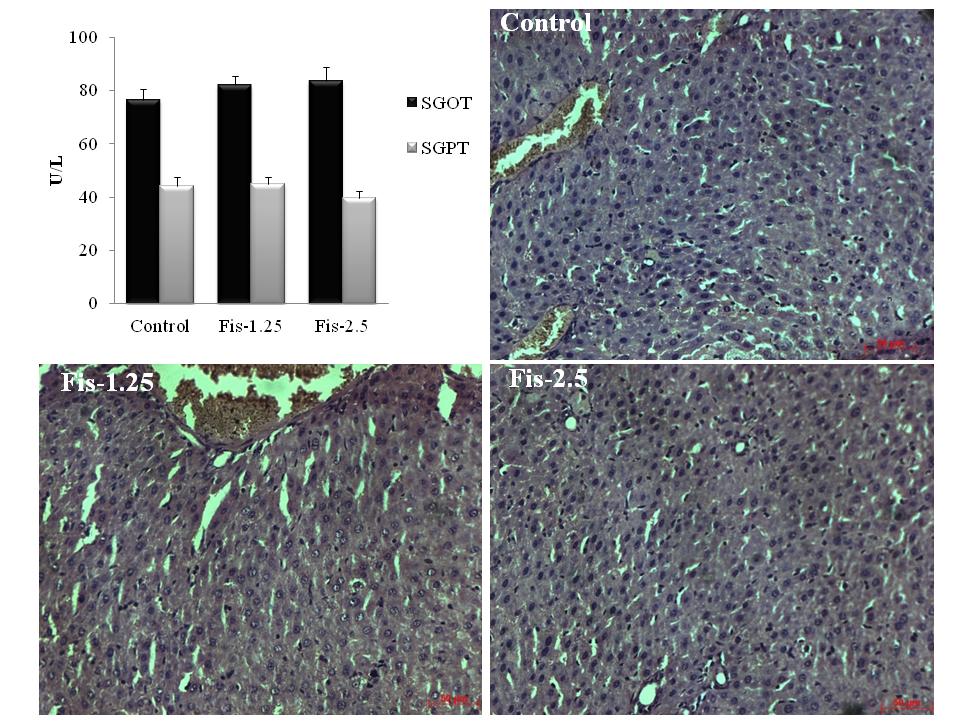

Supplement: Figure S2 — Effect of fisetin itself on serum liver injury biomarkers and liver histology. Intraperitoneal administration of fisetin at two different doses i.e. 1.25 mg/kg and 2.5 mg/kg body weight for 7 consecutive days showing normal serum SGOT (serum glutamic oxaloacetic transaminase) and SGPT (serum glutamic pyruvic transaminase) levels compared to vehicle control rats. Histopathological examination of liver tissue (X200 magnification, scale bar: 50 µm) from vehicle control (Control), fisetin at 1.25 mg/kg (Fis-1.25) and fisetin at 2.5 mg/kg (Fis-2.5) treated rats showing apparently normal histo-morphology. (TIF) [file pone.0105070.s002.tif]
